# Supervised inference of gene-regulatory networks

**DOI:** 10.1186/1471-2105-9-2

**Published:** 2008-01-04

**Authors:** Cuong C To, Jiri Vohradsky

**Affiliations:** 1 Laboratory of Bioinformatics, Institute of Microbiology ASCR, Prague, Czech Republic

## Abstract

**Background:**

Inference of protein interaction networks from various sources of data has become an important topic of both systems and computational biology. Here we present a supervised approach to identification of gene expression regulatory networks.

**Results:**

The method is based on a kernel approach accompanied with genetic programming. As a data source, the method utilizes gene expression time series for prediction of interactions among regulatory proteins and their target genes. The performance of the method was verified using Saccharomyces cerevisiae cell cycle and DNA/RNA/protein biosynthesis gene expression data. The results were compared with independent data sources. Finally, a prediction of novel interactions within yeast gene expression circuits has been performed.

**Conclusion:**

Results show that our algorithm gives, in most cases, results identical with the independent experiments, when compared with the YEASTRACT database. In several cases our algorithm gives predictions of novel interactions which have not been reported.

## Background

In recent years, the inference of protein interaction networks from various sources of data has become an important topic of both systems and computational biology. Protein networks can be represented as a graph with vertices formed by proteins and edges connecting two proteins representing the relationship between them. The interaction can be either direct, where two or more proteins form a functional complex, or indirect – biochemical or regulatory. The biochemical interaction can be, for example, the participation of two enzymes catalyzing two successive biochemical reactions in a pathway. Regulatory interaction represents binding of a transcription factor to a promoter site, which initiates transcription of a particular gene precursor of a protein.

The diversity of protein "interactions" implies also diverse types of data ranging from literature references and sequence database annotations, through biophysical and biochemical data to the data from microarray and proteomics experiments. The type of data predetermines also the type of interaction studied. In this paper we focus on the gene expression networks, where a regulator protein controls expression of a gene precursor of the corresponding protein.

In the last few years, several approaches to the integration of various data sources into one computational framework for inference of network structures have been reported. Particularly suitable for this approach is the concept of kernels [1]. The concept allows for transformation of various data types into kernel matrices, in which each element represents an interaction between two proteins. As an example of use of kernels in computational biology can serve the methods for the prediction of protein-protein interactions from sequences [2,3]. The kernels can be weighted and combined according to the kernel rules, in order to integrate various diverse sources of information, which can be used to predict protein interaction networks. In 2004, Yamanishi *et al.*[4] introduced a supervised approach inspired by spectral clustering for inference of protein networks from multiple data sources, e.g. expression data, protein interaction data, localization data and phylogenetic profiles. They rose an important assumption that interacting proteins (in the general sense mentioned above) share similarities in the data. Based on this assumption, they created a kernel representation of the multisource data of partially known genetic network of *Saccharomyces cerevisiae*. This representation was used as a training set, which was projected onto a conceptual feature space where interacting proteins were grouped. Unknown interactions of the proteins of the training network with other candidate proteins were inferred using canonical correlation analysis. Thus, the authors were able to make new biological inferences about unknown regulatory interactions; they were also able to predict missing enzymes in biochemical pathways.

Another approach using kernels for protein interaction network inference, also based on a kernel matrix completion problem, was proposed by Kato *et al.*[5]. Missing entries, i.e. protein regulatory interactions, other than those used as a training set, were predicted according to the rules derived from the known entries of the training set. In addition, they introduced a system for estimation of the weights assigned to the individual datasets, which differentiated among the levels of influence of the different data types. The obvious drawback of any kernel method for the interaction network prediction is the limitation to the prediction of undirected interactions. However, besides others, the greatest advantage of the kernel methods is the possibility of integrating the data sources of different character into one mathematical framework.

**Figure 1:**
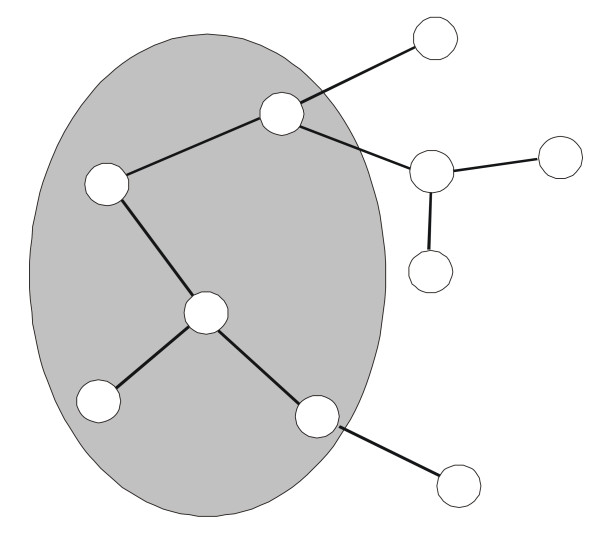
**Principle of prediction of a complete network from a training network (or sub-network, shaded area)**. Vertices represent proteins, edges represent interactions. The training network contains known connections, while the connections outside the shaded area represent connections predicted by extending the rules for training network to other proteins.

The supervised interaction network prediction is based on the assumption that the rules which define connections between proteins in the interaction network can be extended to the proteins of an unknown network. The unknown network is then an expansion of the training network. Figure 1 illustrates this concept.

Our algorithm focuses on identification of missing interactions in a transcriptional regulatory network. We assume that it is possible to project a protein (vertex of the network) to a feature space where the proteins connected in a network are close to each other. Projecting other proteins into the same space can reveal the unknown searched interactions. We assume that the features of an incomplete network can be extended to other, as yet unknown, interactions among proteins of the incomplete network and other proteins (see Figure 1). For the projection of the incomplete network to the conceptual feature space we use its representation by the diffusion kernel combined with kernel principal component analysis (KPCA). For the projection of proteins with unknown interactions with the sub-network, we use their gene expression profiles over time and a function representing relations within the kernel. The function is derived using genetic programming (GP).

Our method was applied to two already known transcriptional regulatory networks of the *Saccharomyces cerevisiae *cell cycle [6] using microarray time series data published by Spellman *et al.*[7]. Other possible interactions, such as protein protein interactions, or indirect biochemical interactions, are in this study ignored; therefore the results obtained here are valid exclusively for regulatory interactions in gene expression.

## Results

From Eq. 15, we see that the projection onto the feature space depends deeply on the approximate functions *f*_*i *_created by GP. Therefore, the control parameters of GP are determined in such way that Eq. 14 is best satisfied using the fitness function defined by Eq. 16.

The details of derivation of the control parameter values can be found in the supplementary materials (see Additional file 1). The parameters used are listed in Table 1.

**Table 1:** A list of control parameters of GP for the search of the kernel approximating function.

Population size:	1000
Maximum generation:	1000
Probability of crossover:	0.90
Probability of reproduction:	0.10
Maximum depth for tree created during run:	10
Maximum depth for initial random tree:	7
Terminal set:	{(*x*_*i*1_, *x*_*i*2_, ..., *x*_*in*_), (*x*_*j*1_, *x*_*j*2_, ..., *x*_*jn*_)}
Function set:	+, -, ×, pow2, pow3, ..., pow10

### Experiments

We used the data from the database of the gene expression profiles of *Saccharomyces cerevisiae *[8] and two protein networks inferred by Lee *et al.*[6] to test the algorithm. Lee et al. in their work identified DNA protein interactions for a set of transcriptional regulators in the genome wide location analysis and inferred interactions for five functional gene groups (cell cycle, metabolism, DNA/RNA/protein biosynthesis and environmental response), and, all together, 106 genes.

The Spellman's database was collected using DNA microarrays and samples collected from growing yeast cultures synchronized by three independent methods: alpha factor arrest (18 time points), elutriation (14 time points), and arrest of a *cdc*15 temperature-sensitive mutant (24 time points). The database is available at [9]. Although both of these datasets are relatively old, they have been extensively studied in the literature on regulatory networks, thus providing an excellent benchmark for model validation and comparison.

**Figure 2:**
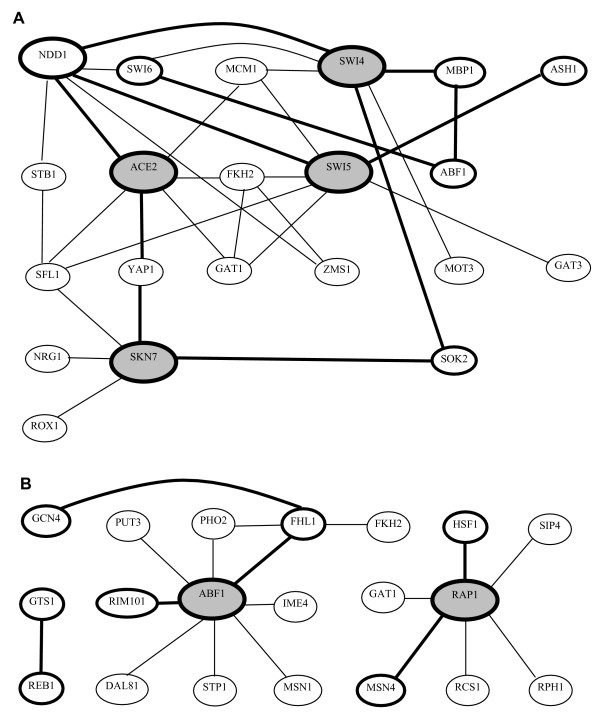
**Interaction networks of genes adopted from the work of Lee et al. [6] with sub-networks (bold) used as a training set**. Shaded nodes represent genes for which the regulatory   interactions were predicted using the algorithm presented here.   A – cell cycle network, B – DNA/RNA/protein synthesis network.

Two protein networks, namely cell cycle and DNA/RNA/Protein biosynthesis identified by Lee et al., served here as a template for comparison with the results of the algorithm we have now presented. Parts of the networks were used as training sets (Figure 2) and the remaining interactions were inferred using the trained algorithm and the genes presented in the work of Lee et al. Such arrangement simulates a situation where only a very limited part of a network is known. In reality, such sub-network can be inferred either from measurements or from a literature surveys. Here we identify the rest of the network using the presented algorithm. In this test example, the complete network is known a priori (we consider the Lee's et al. network as complete for comparison purposes). The prior knowledge allows us to assess the performance of the algorithm by comparison of predicted interactions and interactions inferred from the independent source, the work of Lee et al. For this reason the same set of genes as in the work of Lee at al. was used (the full set of genes is depicted in Figure 7 (see Additional file 1)). The trained algorithm was applied to the expression profiles of these selected genes. The networks inferred by Lee *et al.* and the selected training sub-networks are plotted in Figure 2. The results, i.e. predicted interactions, are listed in Table 2 and Figure 7 (see Additional file 1). For the independent verification of the results of our algorithm and the experimental results of Lee et al., information about the documented and potential interactions among yeast genes and gene products from the YEASTRACT database was used. The YEASTRACT (Yeast Search for Transcriptional Regulators And Consensus Tracking) represents one of the most comprehensive data sources about regulatory interactions in yeast. It is a curated repository which, in the time of publication of this paper, comprised more than 12500 regulatory associations between transcription factors and target genes in *Saccharomyces cerevisiae*, based on more than 900 bibliographic references. It also included the description of 269 specific DNA binding sites for more than a hundred characterized transcription factors.

**Table 2:** Comparison of predictions of regulatory interactions made by the algorithm presented here, results obtained from the paper of Lee et al. [6] and the data from YEASTRACT database for selected genes (cell cycle – ACE2, SKN7, SWI4, SWI5, DNA/RNA/protein synthesis -, ABF1, RAP1).

	Lee *et al.*	This paper	YEASTRACT
ACE2	FKH2	FKH2	FKH2
	GAT1	GAT1	GAT1
	NDD1	NDD1	-
	MCM1	MCM1	MCM1
	SFL1	SFL1	SFL1
	YAP1	-	-
SKN7	ROX1	ROX1	ROX1
	NRG1	NRG1	NRG1
	YAP1	YAP1	YAP1
	SFL1	-	SFL1
	SOK2	SOK2	SOK2
	-	FKH1	FKH1
SWI4	MBP1	-	MBP1
	MCM1	MCM1	MCM1
	MOT3	MOT3	MOT3
	NDD1	-	-
	SOK2	SOK2	SOK2
	SWI4	SWI4	-
	SWI6	SWI6	-
	-	CRZ1	-
	-	DAL82	-
	-	DOT6	-
	-	FZF1	-
	-	GLN3	GLN3
	-	MAL33	-
	-	MSN2	MSN2
	-	RCS1	-
	-	RFX1	RFX1
	-	RGT1	RGT1
	-	RTG3	RTG3
	-	SKO1	SKO1
	-	STP2	-
	-	THI2	-
	-	MDD1	-
SWI5	ASH1	ASH1	ASH1
	FKH2	FKH2	FKH2
	GAT1	-	GAT1
	GAT3	GAT3	GAT3
	MCM1	MCM1	MCM1
	NDD1	NDD1	-
	SFL1	-	SFL1
ABF1	IME4	IME4	IME4
	FHL1	FHL1	FHL1
	MSN1	MSN1	MSN1
	DAL81	DAL81	DAL81
	PHO2	PHO2	PHO2
	PUT3	PUT3	PUT3
	STP1	STP1	STP1
	RIM101	RIM101	RIM101
	-	FZF1	-
	-	HAP2	HAP2
RAP1	GAT1	GAT1	GAT1
	RPH1	RPH1	RPH1
	RCS1	RCS1	RCS1
	MSN4	MSN4	MSN4
	SIP4	SIP4	SIP4
	RAP1	RAP1	-
	HSF1	HSF1	-
	-	SUM1	-
	-	RSF2	-
	-	HAP4	-
	-	GAT3	-
Total	40	56	-
Confirmed by YEASTRACT	32	37	
Not confirmed by YEASTRACT	8	19	
Present in Lee et al.	-	34	

For the cell cycle network, the interactions were inferred using the presented algorithm for the following arbitrarily chosen genes – ACE2, SKN7, SWI4, SWI5, while from the DNA/RNA/Protein biosynthesis network, two genes were chosen – ABF1 and RAP1. Table 2 shows the result of application of the algorithm. Figure 7 (see Additional file 1) shows the graph of interactions for the two networks suggested by Lee et al. and a comparison of the Lee et al. and our results for the selected genes. In the majority of cases, both methods gave similar results. However, our algorithm suggested some more interactions and did not infer some which were predicted by Lee et al. 23 interactions which were not predicted by Lee et al. were suggested by our algorithm. Out of them 8 were confirmed by comparison with YEASTRACT database. Remaining 15 are considered here as false positive. These interactions remain to be confirmed or rejected by future studies.

For the ABF1 gene, our algorithm suggested additional interactions with FZF1 and HAP2. A HAP2 interaction was also confirmed by YEASTRACT. For RAP1, new interactions with SUM1, HAP4 and GAT3 were suggested but not confirmed by YEASTRACT. On the other hand, both methods inferred self control of RAP1 which was not found in the YEASTRACT database. For the cell cycle gene ACE2, our algorithm did not find interactions with YAP1 that were suggested by Lee et al., consistently with the YEASTRACT, which also did not record any regulatory interactions between this gene and ACE2. For SKN7, in contrast with Lee et al., an interaction with SFL1 was not identified by our algorithm. An interaction of SKN7 with FKH1, found in YEASTRACT, was also suggested by our algorithm. For SWI4, 15 additional interactions were suggested by our algorithm, out them 6 were confirmed by YEASTRACT. For the other interactions, predicted by our algorithm, comparison with literature did not confirm them. The interaction with NDD1 suggested by Lee et al. was not found, in accordance with the YEASTRACT database, which also did not report any regulatory interactions between these two genes. For SWI5, our algorithm did not identify interaction with GAT1 and SFL1 reported by Lee et al. These interactions were also not found in YEASTRACT. In contrast both methods suggested an interaction between SWI5 and NDD1 which was not confirmed by YEASTRACT.

We run the algorithm with a smaller training network (Fig. 6 and Table 4 (see Additional file 1)) for each of the control networks mentioned above. Even in this case the algorithm found 40% of genes in agreement with the YEASTRACT database. Overall overlap with the results obtained with the original training network was 37%. The results suggest that – 1. That the algorithm can give acceptable results even in the case of relatively small training network (9 genes for cell cycle and 6 genes for DNA/RNA/protein synthesis networks out of 106 total genes investigated) and – 2. In order to improve the reliability of the results it is advisable to run the algorithm several times for different training networks of similar sizes and select genes which are identified in all or most of the runs. Such approach is common in any evolutionary algorithm based methods.

It can be concluded that the two results (Lee's et al. and ours) obtained by principally two different method gave in most cases identical results. Our algorithm provided suggestion of additional interactions which were not found by the Lee et al's. experiments. Suggested interactions which were not confirmed remain for future verification.

## Discussion and conclusion

Gene control is a time evolving process initiated by binding of a particular regulator (or regulators) to the promoter region of the regulated gene. After that, transcription is initiated and the particular mRNA is synthesized. Recording of the changes of ideally all mRNA amounts over time therefore encodes the information about the regulatory event and, in principle, allows reverse identification of the interactions. In the literature concerning the regulatory network inference, it is frequently assumed that the dependence between protein and mRNA concentration is linear. Therefore, transcriptional control networks have been inferred from gene expression time series. Although this assumption is coarse, the protein concentrations of transcriptional regulators are difficult to measure, and the microarray data are the best available.

Here, we assume that unknown regulatory interactions of proteins sharing similar function can be deduced from a known sub-network (training network) and the gene expression time series. In order to infer the unknown interactions, the known sub-network, that is a part of a complete hypothetical network, was projected to a feature space where the interactions among the nodes of the network are easier to identify. The projection of the sub-network was made using kernels. Functions describing the relations in the sub network kernel rows were identified using genetic programming. The functions are required for projection of the unknown potential regulators to the space of the sub-network. Interactions of the proteins of the training network with the potential regulators were inferred by application of the trained algorithm to the expression profiles of the potential transcriptional regulators.

Here, we used a set of proteins whose interaction network was independently identified previously [6] and compared our results with the Lee's et al. predictions. Both methods were verified by comparison of the results with independent databases of regulatory interactions YEASTRACT. Results show that our algorithm gives, in most cases, results identical with the experiments made by Lee et al., when compared with the YEASTRACT data, our algorithm suggests additional interactions which were not found in literature. Most of the differences between our results and the results of Lee et al. are concentrated into two genes (SWI4, RAP1), when our algorithm gives predictions of interactions which are neither listed in the work of Lee et al. nor in the YEASTRACT database (if we exclude these genes agreement of our results with YEASTRACT is 92%, 88% for Lee et al.). We have made a Medline search in the attempt to find other possible literature references, but among the thousands of records, we were not able to find those confirming the predicted interactions. Here we consider them as false positives. Nonetheless, as the method proved to be very efficient in the prediction of gene control, they can also be considered as suggestions for further experimental verification. It is necessary to emphasize that the networks reconstructed using kernel methods are in general undirected.

The results of the network reconstruction generally depend on the size of the training network; the bigger it is the more reliable are the predictions. Also, as for the other evolutionary algorithms, the reliability of prediction can be increased by running the algorithm several times for different training networks.

Although this work utilizes the time series and a diffusion kernel, other data sources, such as promoter sequence similarity, literature information and others can be used to create individual kernels and combine them into a single kernel, using the kernel combination rules. This representation can be used further for training of the algorithm and for the inference of additional interactions.

In recent years, a repository of gene expression profiles recorded using either microarrays or the proteomic approach during time evolving processes, has grown rapidly, therefore, large amounts of data containing the information about the regulatory interactions controlling the given process are available. The algorithm suggested here can serve for their identification.

## Methods

### Kernel representation

Inference of complex regulatory networks from experimental data is a combinatorial problem which has been addressed by various optimization techniques (for review see [10,11]). Finding the solution to this problem generally is difficult and can lead, for bigger networks, to many equivalent solutions. Kernel representation simplifies the definition of the interaction within the network to a positive definite kernel matrix, with each element proportional to the strength of the interaction between the regulator and a gene precursor of the protein.

A convenient representation of an interaction network is the diffusion kernel. The diffusion kernel was derived from an analogy with diffusion of heat, or diffusion of a compound in a diluted solution (Ficks law). A formula for the diffusion kernel can be derived from the idea of a random walk [12]

(1) $$K_{\beta }=\underset{s\to \infty }{\lim}{\left(I+\frac{\beta L}{s}\right)}^{s}s\in \mathbb{N}$$

for a given *β *and identity matrix *I*, where

(2) $$L_{ij}=\{\begin{array}{ll} 1 & \text{if i}∼\text{j}\\ -\gamma_{i} & \text{if i}=\text{j}\\ \text{0} & \text{otherwise} \end{array}$$

is called "graph Laplacian" and *γ*_*i *_is the degree of vertex *i*, i.e. the number of edges incident at *i*. By analogy with the exponentiation of real numbers, the limit (1) is called matrix exponentiation of L

(3) $$e^{\beta L}=\underset{s\to \infty }{\lim}{\left(I+\frac{\beta L}{s}\right)}^{s}$$

which can be expanded to the power series

(4) $$e^{\beta L}=I+\beta L+\frac{\beta^{2}}{2}L^{2}+\frac{\beta^{3}}{6}L^{3}+...$$

If we compute normalized eigenvectors *v*_1_*, v*_2_,..., *v*_*n *_and corresponding eigenvalues *λ*_1_, *λ*_2_,..., *λ*_*n *_of *L*, then, according to the orthogonality, we get

(5) $$L^{s}={\left(\sum_{i=1}^{n}v_{i}\lambda_{i}v_{i}^{T}\right)}^{s}=\sum_{i=1}^{n}v_{i}\lambda_{i}^{s}v_{i}^{T}$$

and according to 4

(6) $$\begin{array}{l} e^{\beta L}=I+\left(\sum_{i=1}^{n}v_{i}\lambda_{i}^{s}v_{i}^{T}\right)+\left(\sum_{i=1}^{n}v_{i}\frac{{\left(\beta \lambda_{i}\right)}^{2}}{2}v_{i}^{T}\right)+\ldots =\\ =\sum_{i=1}^{n}v_{i}e^{\beta \lambda_{i}}v_{i}^{T} \end{array}$$

The Eq. 6 was used to compute the kernel *K*_*β*_.

An example of the diffusion kernel for a simple network is given in Figure 1 (see Additional file 1).

### Supervised prediction

**Figure 3:**
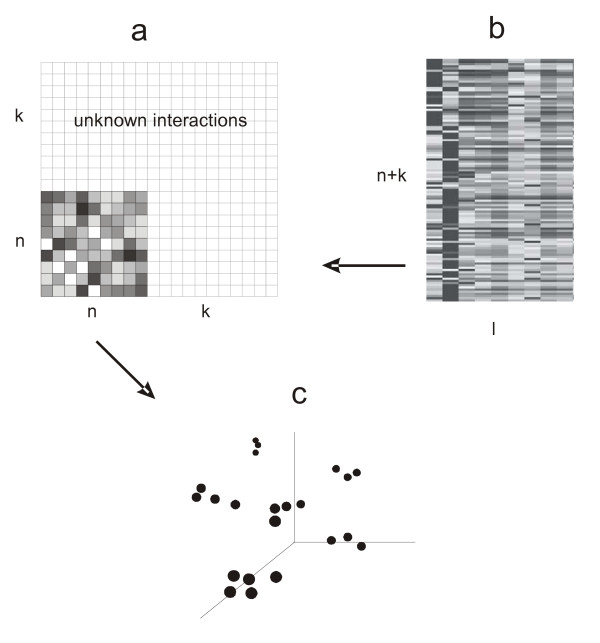
**Symbolic representation of supervised inference of protein interaction network**. Filled area of panel **a **represent known part of the network to be inferred. **b **– time series of microarray or proteomic experiment. Both data are mapped onto a common feature space **c **where the interaction of the proteins is inferred from the known interactions shown in panel **a**.

The supervised approach to protein interaction prediction suggested by Yamanishi *et al.*[4] and used also here, is illustrated in Figure 3. We would like to infer missing network structure (Figure 3a) from experimental time series (Figure 3b), when we already know a part of the network. This situation is depicted in Figure 3a, where we assume that part of the interaction network of *n *proteins is known. This network can be characterized by a diffusion kernel.

Identification of the missing proteins and their interactions means finding a feature space where whenever **x**_i _interacts with **x**_j_, the mapping *f *(**x**_i_) is similar to *f *(**x**_j_). Yamanishi [4], based on the previous work of Bach and Jordan [13], used the canonical correlation analysis (CCA) for generalization of the kernel features and derived an equation

(7) $$f^{\left(l\right)}(x)=\sum_{k=1}^{n}\alpha_{1}^{\left(l\right)}(x_{k})K(x_{k},x)$$

where *f*(**x**) maps vector **x **into a feature space defined by the *l*-th solution *α *of CCA (for details see [4]). *K *(**x**_*k*_, **x**) is a kernel computed from genomic data.

### Kernel PCA

The kernel PCA was originally used in spectral clustering [14]. The idea of spectral clustering is a projection of the data matrix onto a feature space where clusters could be identified using a classical clustering algorithm. The feature space is defined by eigenvectors of the kernel principal component analysis (KPCA).

The principle of KPCA is to map an input space to a different high dimensional space. Let **X **= {**x**_1,_..., **x**_*m*_}, where **x**_*k *_∈ ℜ^*n*^, is a vectorial representation of *k*-th protein profile of a total of *m *proteins. We assume that these vectors are centered, that is. Σ **x**_*k *_= 0. The basic concept of KPCA is first to map the input data **X **into a feature space Φ via a nonlinear mapping *φ *(·) and then perform a linear PCA in Φ [1]. Classical PCA [15] diagonalizes the covariance matrix **C **by

(8) **Ce = λe**

with eigenvectors **e **and eigenvalues λ. Let

(9) **Φ **= [*φ*(**x**_1_)|...|*φ*(**x**_*m*_)]

be the image of **X **in the feature space. Assuming that the mapped data are centered,

(10) **K **= **Φ**^T ^**Φ**, with *K*_ij _= *φ *(**x**_*i*_)·*φ*(**x**_*j*_).

Solving Eq. 8 for **K **given by Eq. 10, we have

(11) **e **= **Φα**

For a vector **x**, the projection on principal component *y *corresponding to eigenvector **e **is computed by

(12) $$y=e^{\text{T}}\phi (x)=\alpha^{\text{T}}\Phi^{\text{T}}\phi (x)=\sum_{i=1}^{m}\alpha_{i}\phi (x_{i})\cdot \phi (x)$$

The dot-product matrix **K **can be computed by choosing a kernel *k*(**x**, **y**) such that *k*(**x**_*i*_, **x**_*j*_) = *φ*(**x**_*i*_). *φ*(**x**_*j*_) = *K*_*ij *_(this is referred to as a kernel trick). Therefore, Eq.12 can be rewritten as:

(13) $$y=\sum_{i=1}^{m}\alpha_{i}k(x_{i},x)$$

If *k*(**x**_i_, **x**) is a linear kernel, the KPCA converts to a classical PCA.

Choice of appropriate kernels allows for nonlinear mapping of **X **into the feature space which can be more suitable for the given task than the original space. Moreover the principal components are orthogonal and thus uncorrelated, and the first principal components carry most of the variance of the dataset, as in the classical PCA. Therefore, only the first few principal components can be used for the cluster identification.

The ultimate goal of the network identification algorithm is the identification of a complete regulatory network with all interactions. Unfortunately, we never know in advance all proteins forming the nodes of the network. On the other hand, we usually know some part of this hypothetical network. Therefore, all proteins which can play some role in transcriptional regulation have to be considered as possible members of the network. The basic idea of our algorithm is similar to the spectral clustering i.e. the projection of each protein onto a feature space where the connected proteins have similar coordinates. Conversely, the similarity of the projection of proteins with unknown connections can then be used to deduce the connections among them.

Knowing only part of the network, not all possible network members can readily be projected onto the feature space. However, the known part of the hypothetical network can be represented by the diffusion kernel and projected to the feature space using Eq. 13. Projection of other proteins to the same space can reveal their unknown connections with proteins of the known part of the network (see Figure 3).

Let TP = {**x**_*i *_∈ R^*n*^, *i *= 1..*m*} be a vectorial representation of *m *proteins forming the known part of the complete hypothetical network. Its graphical representation will be called a training graph. Let UP be a set of *n *proteins which possibly can be a part of the complete hypothetical network, with **z**_i _∈ R^*n*^, *i *= 1..*n *representing their expression profiles. These proteins can be either transcriptional regulators or other proteins which we expect to play a role in transcriptional control. Knowing the connections among the proteins of the training graph, we can directly calculate the diffusion kernel *K*. Eq 13 is then used to project the proteins of the training graph onto the feature space. Extending the features of the training graph allows the identification of connections for the proteins from UP. The extension of the network requires calculation of the kernel values for the proteins from UP. Due to the principles of the diffusion kernel, the extension of the training graph kernel to the other proteins is not readily possible. The diffusion kernel values *k*(**x**, **y**) can only be computed for two proteins, **x **and **y, **with already known connections. We cannot calculate directly *k*(**x**, **y**) for proteins with unknown connections, i.e. the proteins which are not part of the training graph. Consequently, we cannot directly project the other proteins onto the feature space and therefore we cannot identify their connections.

In order to solve this problem the diffusion kernel *k*(**x**_i_, **x**) of the training graph can be viewed as a function of two variables that satisfy *f*(**x**_i_, **x**) ≈ *k*(**x**_i_, **x**). Therefore, first a table value of the diffusion kernel (Table 3) for the proteins forming the training graph, is computed and the appropriate functions with

**Table 3:** Diffusion kernel values of graph and approximate functions

*k*(**x**_*i*_, **x**_*j*_)	Diffusion kernel values				approximate functions
		
	**x**_1_	**x**_2_	...	**x**_*m*_	
**x**_1_	*k*(**x**_1_, **x**_1_)	*k*(**x**_1_, **x**_2_)	...	*k*(**x**_1_, **x**_*m*_)	*f*_1_(**x**_1_, **x**_j_)
**x**_2_	*k*(**x**_2_, **x**_1_)	*k*(**x**_2_, **x**_2_)		*k*(**x**_2_, **x**_*m*_)	*f*_2_(**x**_2_, **x**_j_)
...	...	...	...	...	...
**x**_*m*_	*k*(**x**_*m*_, **x**_1_)	*k*(**x**_*m*_, **x**_2_)		*k*(**x**_*m*_, **x**_*m*_)	*f*_*m*_(**x**_*m*_, **x**_j_)

(14) *f*_*i*_(**x**_*i*_, **x**_*j*_) ≈ k(**x**_*i*_, **x**_*j*_) (*i*, *j *= 1..*m*)

are identified. Then Eq. 12 can be rewritten to the form:

(15) $$y=\sum_{i=1}^{m}\alpha_{i}f_{i}(x_{i},z)$$

for expression profile **z **of a protein with unknown connection to the training graph. After we have the set of functions approximating the diffusion kernel and the expression profiles **z **we can calculate the kernel values for the proteins with unknown connections to the known part of the network. After that, we use the KPCA to project them onto the feature space of the training graph. Thus, identification of unknown protein connections converts to an identification of a set of functions approximating the diffusion kernel and performing KPCA for the unknown proteins. The whole scheme is depicted in Figure 5 (see Additional file 1).

### Prediction

Proteins of the training graph are projected into the feature space using the diffusion kernel and Eq. 13. Using Eq. 14 and 15 other proteins with unknown but probable interactions with the known part of the network are projected onto the same feature space. The prediction is then computed in the feature space. Let A = {**x**_*i*_∈ Φ, *i *= 1..*j*} be a set of proteins from the training graph which have known direct connections to protein **x**_*k*_. Let *d*_max _= max {*d*(**x**_*i*_, **x**_*k*_), **x**_*i*_, **x**_*k*_∈ A} be the maximum distance (Euclidean) from **x**_*k *_to all proteins of A. If a protein with an expression profile **z **with unknown connection to the proteins of the training graph has a distance from **x**_*k *_*d*(**z**, **x**_*k*_) ≤ *d*_max_, then **z **is predicted to have a direct connection to **x**_*k*_.

### Genetic programming

Genetic programming (GP) is an extension of genetic algorithm methods which was thoroughly discussed by Koza [16]. During the training process the GP algorithm discovers relationships between the input variables of the training set using the rules (operators). These rules can contain logical relationships, mathematical operators or any other defined relationships. The rules combine variables to an output, e.g. operation a + b = c is represented by the rule "+" and input variables a and b with the output c. c can be again combined with other variables or results of other operations on variables to a tree-like sequence of operations. Training is performed to find a tree which combines the variables and operators and satisfies best the output conditions which are defined by the so called fitness function. As with any evolutionary computing methods, the optimization of the trees is done by means of operations of reproduction and crossover.

The initial population of trees is created randomly, using the variables and a predefined set of operators. The operations of crossover and reproduction, when parts of the trees are randomly combined or simply copied, create a new generation. Ability of the trees to perform a requested task is evaluated in each generation by means of the fitness function. The control parameters of GP are: the maximum number of generations, probability of crossover, probability of reproduction, maximum depth of the tree created during the run, maximum depth of the initial random tree, and the allowed set of operators (function set).

It has been shown that such a scheme leads to improvement in the fitness value with an increasing number of generations [16]. The iterations are repeated until the criteria for fitness are satisfied or a preset number of iterations is reached. The resulting specific program (tree) is then applied to perform the task coded in the program with a given set of data.

Here we used genetic programming to find a set of functions *f*(**x**_i_, **x**) satisfying Eq. 14. This allows us to define a fitness function as

(16) $$fitness=\sum_{j=1}^{m}\left|f_{i}(x_{i},x_{j})-k(x_{i},x_{j})\right|$$

which was used during the GP function search.

## Authors' contributions

CT designed the algorithm and made the computations. JV conceived of the study, and participated in its design and coordination and wrote the manuscript. All authors read and approved the final manuscript.

## Supplementary Material

Additional file 1 To Vohradsky supplementary materials. Supplementary figures and tables. Click here for file
