# Supplementary material for: Supervised inference of gene-regulatory networks
Source: BMC Bioinformatics. 2008 Jan 4;9:2. doi: 10.1186/1471-2105-9-2 (PMC2266705; doi:10.1186/1471-2105-9-2)

Supervised inference of gene regulatory networks

Cuong C. To, Jiri Vohradsky*

Supplementary materials

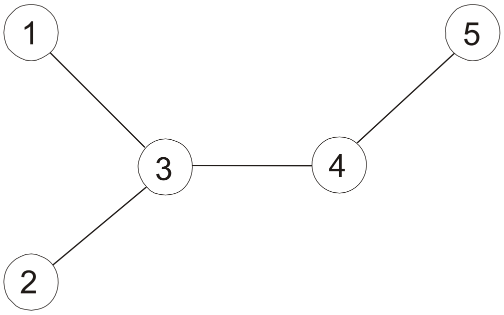

$$L = \begin{pmatrix} 1 & 0 & -1 & 0 & 0 \\ 0 & 1 & -1 & 0 & 0 \\ -1 & -1 & 3 & -1 & 0 \\ 0 & 0 & -1 & 2 & -1 \\ 0 & 0 & 0 & -1 & 1 \end{pmatrix} \qquad K = \begin{pmatrix} 0.49 & 0.12 & 0.23 & 0.10 & 0.03 \\ 0.12 & 0.49 & 0.23 & 0.10 & 0.03 \\ 0.23 & 0.23 & 0.24 & 0.17 & 0.1 \\ 0.1 & 0.1 & 0.17 & 0.31 & 0.30 \\ 0.03 & 0.03 & 0.01 & 0.30 & 0.52 \end{pmatrix}$$

Figure 1. Matrix L (Eq.2) and corresponding diffusion kernel derived from a simple network graph with 5 nodes. Kernel K was computed using β=1.

Control parameters of GP

The control parameters of GP were optimized using two arbitrary artificially created interaction graphs (Figure 2).

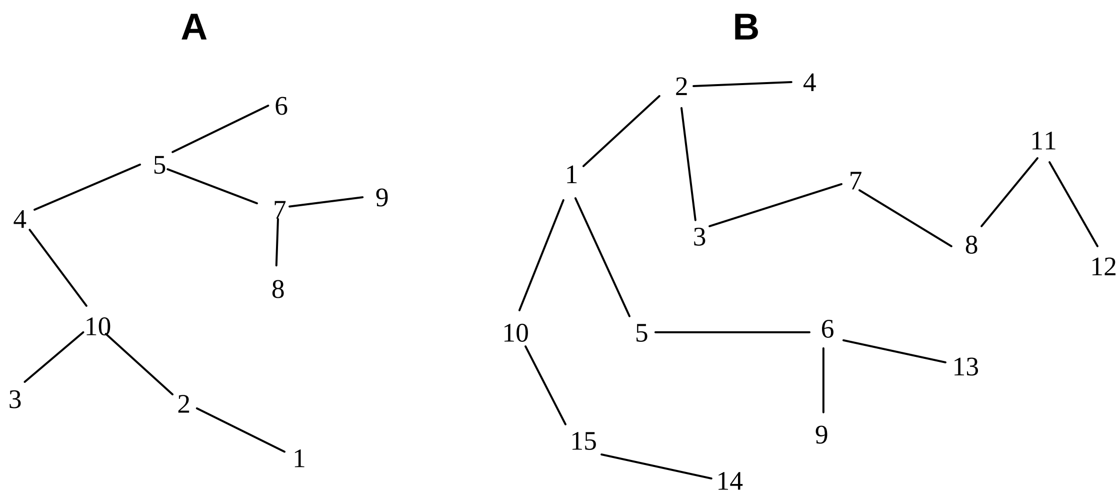

Figure 2. Two artificial networks used for determination of parameters of genetic programming.

In order to determine the optimal population size and the number of generations, the probability of crossover was set to 0.9 and size of the population and the number of generations were varied from 500 to 1000 (for population size) and 500 to 1100 (for number of generations).For each combination of the two parameters and both networks the fitness values were computed. Results are given in Tables 1,2 and Figures 3,4. The tables and the graphs show that the fitness value decreases with increasing number of generations and population size. Minimal value is reached for population size of 1000 and number of generations 1000-1100 for which the fitness value does not improve with increasing number of generations. Therefore the values of population size and number of generations were both set to 1000.

The influence of probability of crossover on the fitness was examined for already determined values of population size and number of generations equal to 1000. The probability of crossover was varied from 0.5 to 0.9. Table 2 and Figure 2 of supplementary materials show the influence of the probability of crossover on the value of fitness. Inspection of the graph shows that fitness value decreases with increasing probability of crossover with biggest improvement between 0.6 and 0.7 and with minimum at 0.9. Based on these results we chose a value of 0.9. The value of probability of reproduction did not exhibit marked influence (data not shown) and was arbitrarily set to 0.1. All control parameters of GP used are listed in Table 3.

Table 1. Fitness value for two artificial networks of Figure 2 for different values of population size and number of generations. The values are plotted in Figure 3.

a – network A.

Fitness values		Population					
		500	600	700	800	900	1000
r a	500	0.099618	0.090997	0.07941	0.073576	0.060897	0.061323

	600	0.099375	0.086829	0.076924	0.07102	0.058863	0.061095
	700	0.098908	0.084902	0.07519	0.06586	0.058322	0.056112
	800	0.096053	0.078657	0.07257	0.060204	0.056636	0.054738
	900	0.095635	0.077444	0.069137	0.059919	0.052451	0.041684
	1000	0.086109	0.075776	0.064811	0.057107	0.051479	0.029934
	1100	0.083869	0.069841	0.06335	0.057021	0.040233	0.028829

b – network B

Fitness values		Population					
		500	600	700	800	900	1000
Generation	500	0.17361	0.158961	0.138494	0.132462	0.124732	0.113849
	600	0.158758	0.152739	0.134715	0.129576	0.116904	0.114059
	700	0.158378	0.142356	0.131643	0.125228	0.111505	0.104619
	800	0.158043	0.138654	0.129423	0.124554	0.109635	0.101857
	900	0.154255	0.134164	0.127993	0.116447	0.103831	0.090344
	1000	0.148454	0.133755	0.120551	0.112711	0.101611	0.065257
	1100	0.146273	0.12318	0.120682	0.110477	0.102646	0.061258

Figure 3. Fitness value for two artificial networks of Figure 3 (main document) for different values of population size and number of generations.

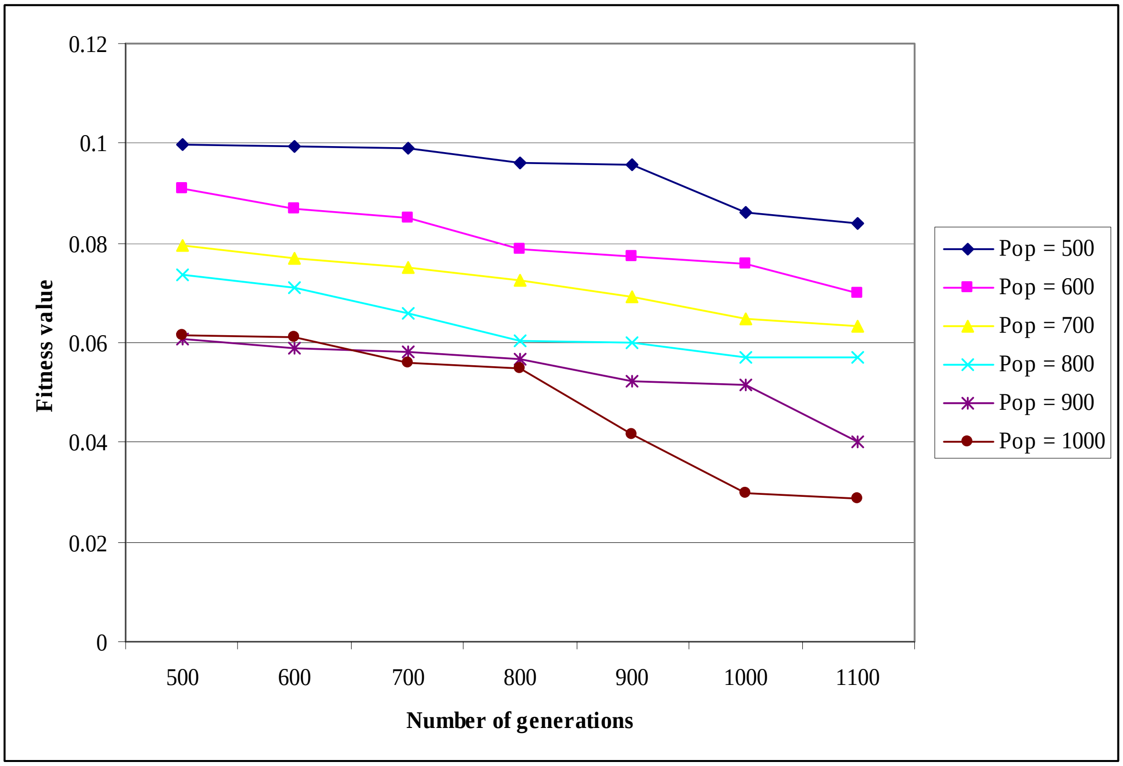

Network A

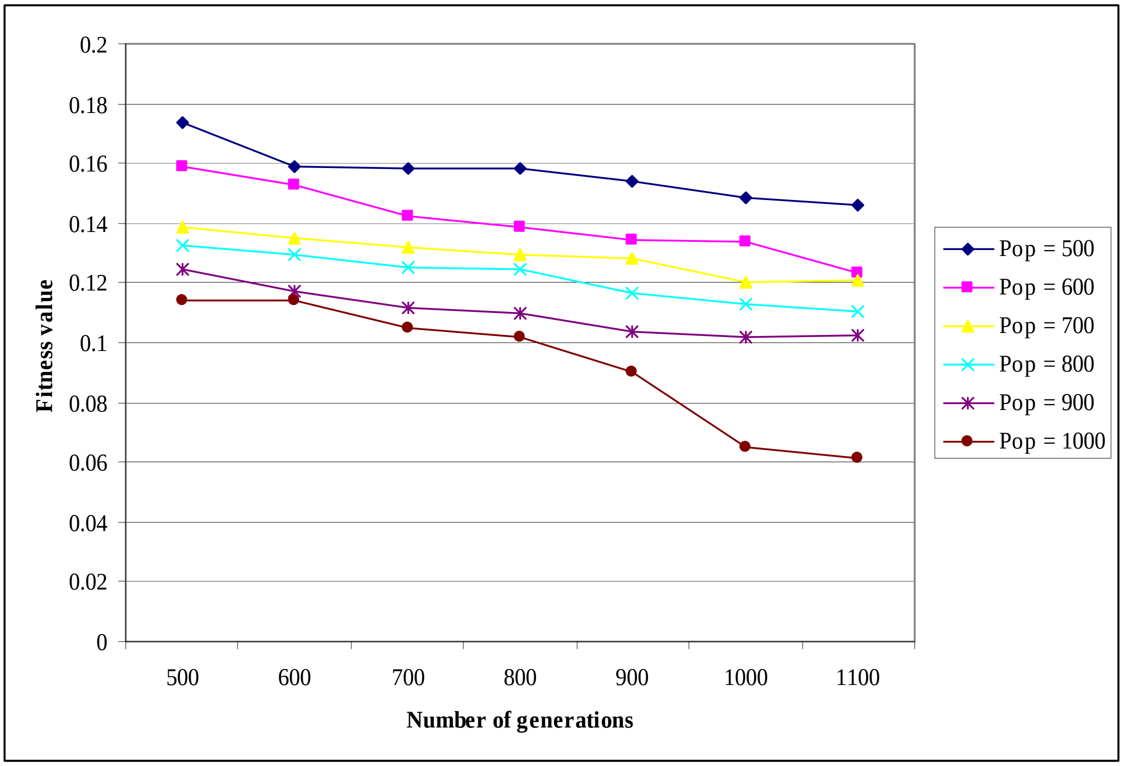

Network B

Fitness values	Probability of crossover				
	0.5	0.6	0.7	0.8	0.9
Graph 1	0.041321	0.036887	0.034767	0.033633	0.029934
Graph 2	0.097347	0.098583	0.071921	0.077402	0.065257

Table 2. Fitness value for two artificial networks of Figure 2 for different values of probability of crossover. Population size and number of generations were both set to 1000. The values are plotted in Figure 4.

Figure 4. Fitness value for two artificial networks of Figure 2 for different values of probability of crosover.

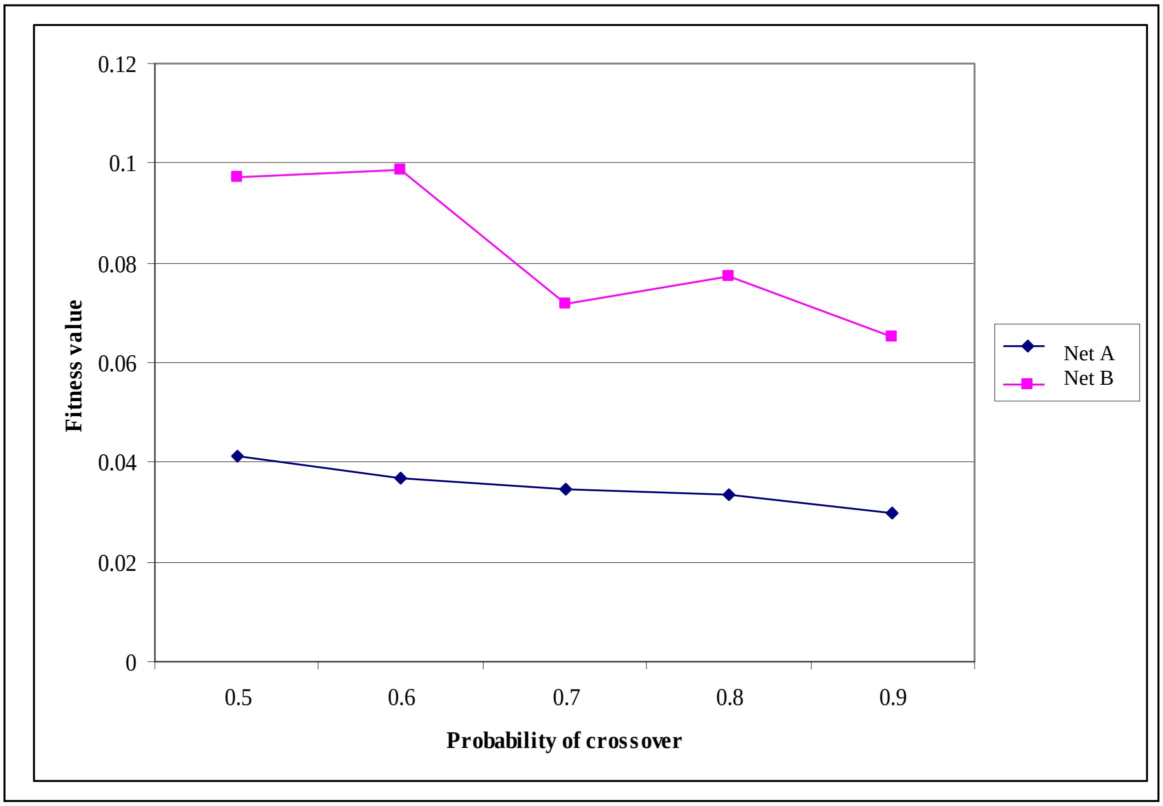

Population size:	1000
Maximum generation:	1000
Probability of crossover:	0.90
Probability of reproduction:	0.10
Maximum depth for tree created during run:	10
Maximum depth for initial random tree:	7
Terminal set:	$\{(x_{i1}, x_{i2}, \ldots, x_{in}), (x_{j1}, x_{j2}, \ldots, x_{jn})\}$
Function set:	+, -, ×, pow2, pow3, …, pow10

Table 3. A list of control parameters of GP for the search of kernel approximating function.

Figure 5. A flow chart of supervised projection of proteins onto a feature space.

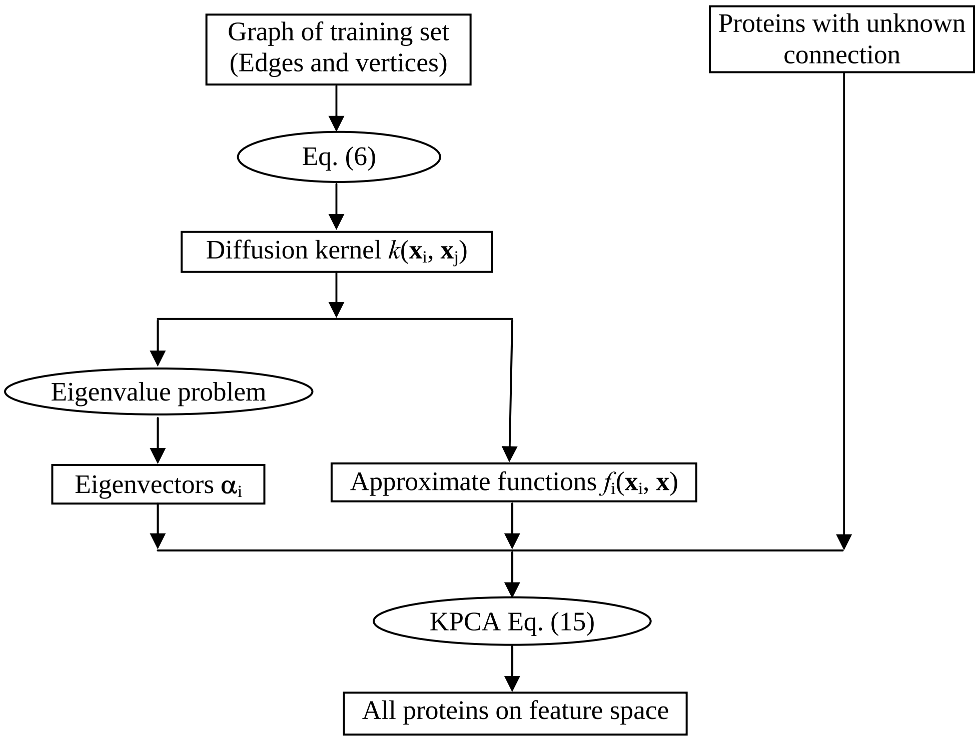

Figure 6 - Interaction networks of genes adopted from the work of Lee et al. (Lee, et al., 2002) with sub-networks (bold) used as a training set for the results in Table 4. Shaded nodes represent genes for which the regulatory interactions were predicted using the algorithm presented here. A – cell cycle network, B – DNA/RNA/protein synthesis network.

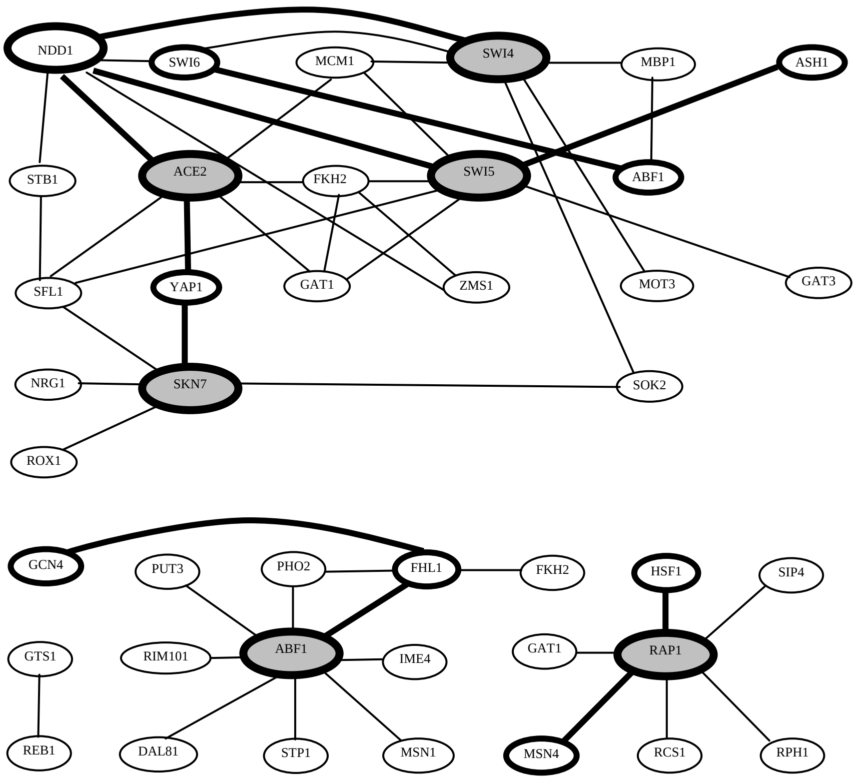

Table 4. Comparison of predictions of regulatory interactions made by the algorithm presented here, results obtained from the paper of Lee et al. (Lee, et al., 2002) and the data from YEASTRACT database for selected genes (cell cycle - ACE2, SKN7, SWI4, SWI5, DNA/RNA/protein synthesis – , ABF1, RAP1) for the training network in Figure 6.

	Lee et al.		YEASTRACT
ACE2	FKH2	-	FKH2
	GAT1	-	GAT1
	NDD1	-	-
	MCM1	MCM1	MCM1
	SFL1	-	SFL1
	YAP1	-	-
SKN7	ROX1	ROX1	ROX1
	NRG1	-	NRG1
	YAP1	-	YAP1
	SFL1	-	SFL1
	SOK2	-	SOK2
	-	STP2	-
	-	STE12	STE12
	-	HIR2	-
	-	DIG1	-
	-	GCR1	GCR1
	-	RLM1	RLM1
	-	CUP9	-
	-	GAL4	-
	-	FHL1	-
SWI4	MBP1	MBP1	MBP1
	MCM1	MCM1	MCM1
	MOT3	MOT3	MOT3
	NDD1	-	-
	SOK2	SOK2	SOK2
	SWI4	-	-
	SWI6	-	-
	-	MAL33	-
	-	RTG3	RTG3
	-	STP2	-
	-	HAP3	HAP3
	-	ADR1	-
	-	SUM1	-
	-	STE12	-
	-	SKN7	-
	-	BAS1	-
	-	MSS1	-
	-	CRZ1	-
	-	MET4	-
SWI5	ASH1	-	ASH1

	FKH2	-	FKH2
	GAT1	-	GAT1
	GAT3	-	GAT3
	MCM1	MCM1	MCM1
	NDD1	NDD1	-
	SFL1	-	SFL1
	-	SOK2	SOK2
	-	HIR2	-
NDD1	STB1	-	-
	SWI4	-	SWI4
	SWI5	SWI5	-
	SWI6	-	SWI6
	ZMS1	-	-
	-	HAP3	-
	-	SMP1	-
	-	DOT6	-
	-	IME4	-
	-	FKH1	FKH1
	-	RSF2	-
	-	HAP4	-
	-	ASH1	ASH1
	-	ACE2	-
	-	ARG81	-
	-	SOK2	-
	-	STB1	-
ABF1	IME4	-	IME4
	FHL1	FHL1	FHL1
	MSN1	MSN1	MSN1
	DAL81	DAL81	DAL81
	PHO2	-	PHO2
	PUT3	-	PUT3
	STP1	-	STP1
	RIM101	RIM101	RIM101
	-	FZF1	-
	-	HAP2	HAP2
	-	HAP3	HAP3
	-	REB1	-
	-	MBP1	MBP1
	-	NRG1	NRG1
	-	SUM1	-
	-	SWI4	SWI4
	-	GAT1	-
	-	OTU1	-
	-	MIG1	-
	-	AFT1	-
	-	CBF1	-
	-	PHD1	-
	-	HIR2	-
RAP1	GAT1	GAT1	GAT1
	RPH1	RPH1	RPH1
	RCS1	-	RCS1
	MSN4	MSN4	MSN4
	SIP4	-	SIP4

	RAP1	-	-
	HSF1	-	-
	-	SUM1	-
	-	HAP4	-
	-	MBP1	
	-	NRG1	NRG1
	-	OTU1	-
	-	MIG1	-
	-	AFT1	-
	-	HAP2	-
	-	YAP3	YAP3
	-	RIM101	-
	-	YAP1	YAP1
	-	DAL82	-
	-	RTG1	RTG1
	-	INO4	-
	-	HIR2	-
	-	AZF1	AZF1
Total	40	82	-
Confirmed by YEASTRACT	32	32	-
Not confirmed by YEASTRACT	8	50	-
Present in Lee et al.	-	16	

Figure 7. Comparison of interactions infered by Lee et al. and by the algorithm from this paper for 2 regulatory networks.

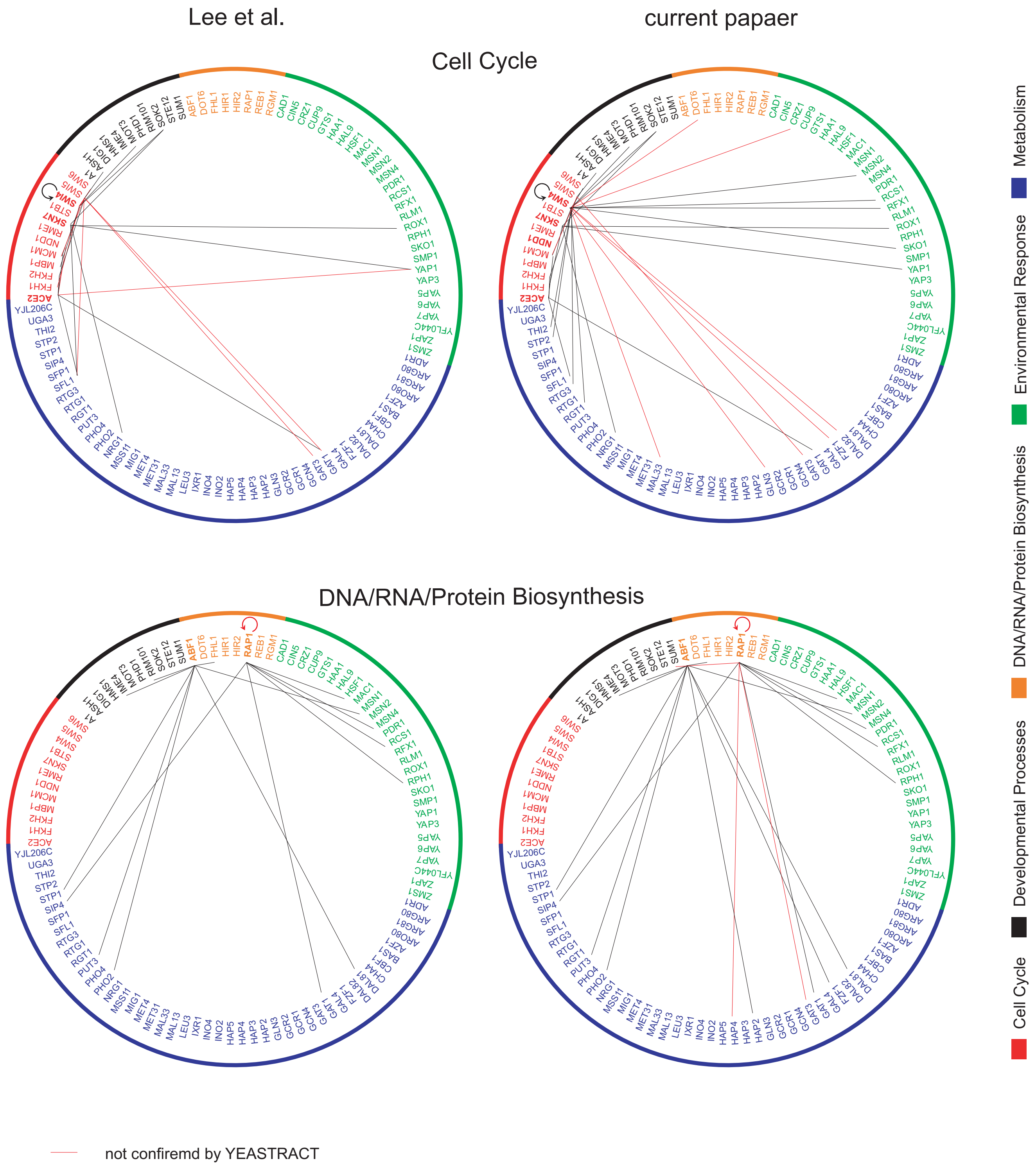

Supplement: Additional file 1 — To Vohradsky supplementary materials. Supplementary figures and tables. [file 1471-2105-9-2-S1.pdf]
